# The Biology of Eosinophils and Their Role in Asthma

**DOI:** 10.3389/fmed.2017.00093

**Published:** 2017-06-30

**Authors:** Claire N. McBrien, Andrew Menzies-Gow

**Affiliations:** ^1^Imperial College London, London, United Kingdom; ^2^Royal Brompton and Harefield NHS Foundation Trust, London, United Kingdom

**Keywords:** eosinophils, asthma, IL-5, eosinophil receptors, respiratory tract infections, asthma exacerbation

## Abstract

This review will describe the structure and function of the eosinophil. The roles of several relevant cell surface molecules and receptors will be discussed. We will also explore the systemic and local processes triggering eosinophil differentiation, maturation, and migration to the lungs in asthma, as well as the cytokine-mediated pathways that result in eosinophil activation and degranulation, i.e., the release of multiple pro-inflammatory substances from eosinophil-specific granules, including cationic proteins, cytokines, chemokines growth factors, and enzymes. We will discuss the current understanding of the roles that eosinophils play in key asthma processes such as airway hyperresponsiveness, mucus hypersecretion, and airway remodeling, in addition to the evidence relating to eosinophil–pathogen interactions within the lungs.

## Introduction

The three main processes responsible for the clinical features of asthma are well recognized: bronchoconstriction, mucus hypersecretion, and airway inflammation. However, the underlying pathophysiology responsible for these processes is complex and nuanced, involving multiple cell types and cytokines (1). Furthermore, the activity and clinical impact of each cellular and subcellular component varies considerably between individuals and can change over time, as well as in response to drug therapy and environmental/lifestyle influences.

Among these myriad cellular interactions and this extremely heterogeneous patient group, it is possible to identify certain key cells that are commonly involved—of which, arguably, the eosinophil is the most important.

Eosinophil precursors originate in the bone marrow and following differentiation traffic to the lungs (among other sites) *via* the bloodstream (2). While high concentrations of circulating eosinophils are often measured in asthmatic patients, of more clinical relevance is the lung tissue eosinophilia that is also frequently present.

The phenotype of “severe eosinophilic asthma” refers to a subgroup of asthmatic patients with evidence of eosinophilia that often require high maintenance doses of oral corticosteroids to maintain reasonable disease control. The notoriously non-specific mechanisms of action of corticosteroid therapy give rise to numerous well-documented adverse effects (3), which have driven decades of research focused on the development of targeted anti-eosinophil drug therapies. In order to understand how to better assist this group of patients, who currently have an unmet clinical need, it is helpful to understand the eosinophil itself, and the role that it plays in asthma. Targeted anti-eosinophil therapies will be touched upon but will be covered in greater detail by other reviews in this Research Topic.

## Eosinophil Cell Structure

**Figure 1:**
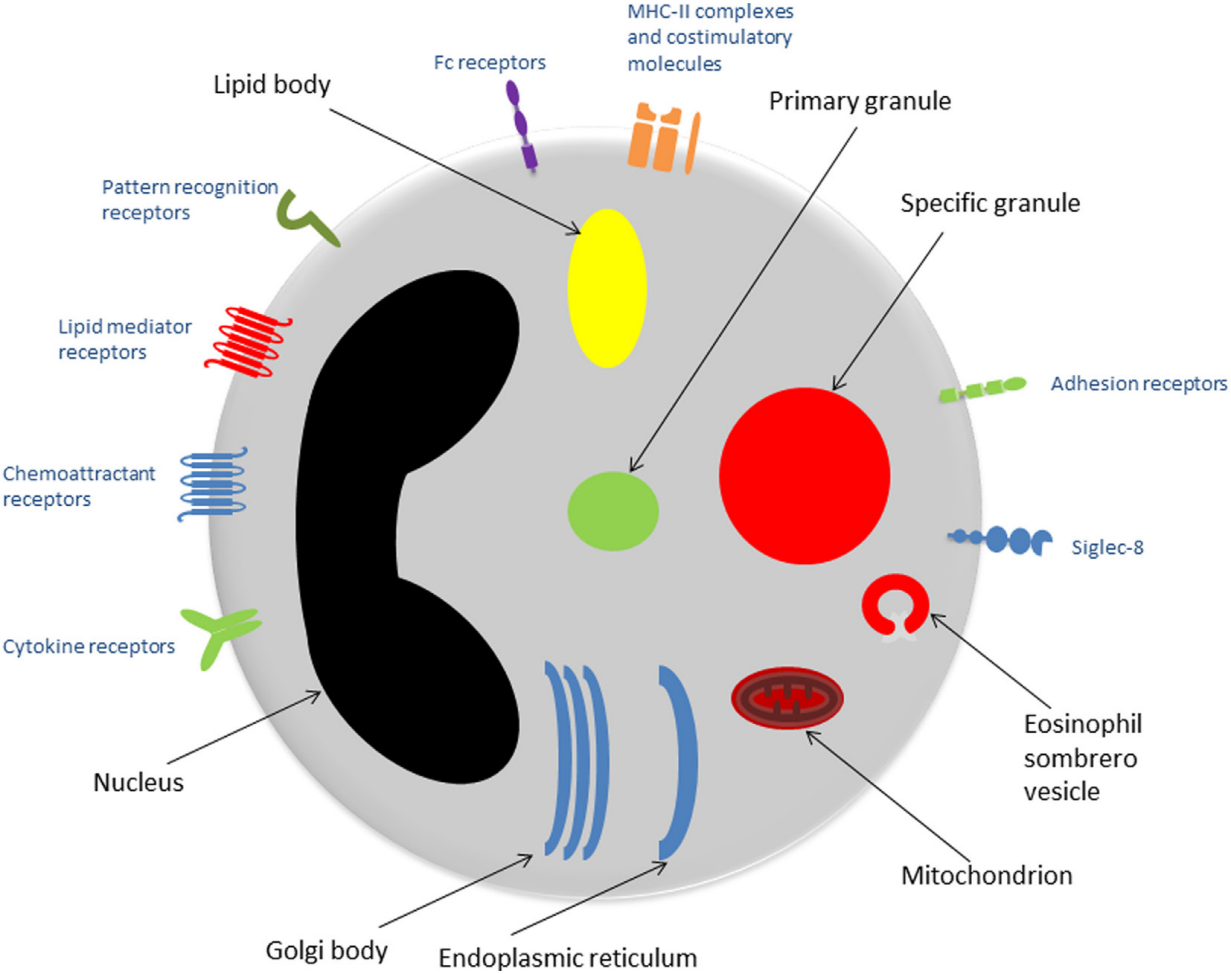
Eosinophil ultrastructure. Schematic representation of an eosinophil showing the major organelles (black labels) and cell surface receptors (blue labels). Abbreviation: MHC-II, major histocompatibility complex-II.

Eosinophils are granulocytes, typically measuring 10–16 µm in diameter. They possess segmented (usually bi-lobed) nuclei and their nucleus: cytoplasm ratio is approximately 30%. Eosinophils stain with acidophilic dyes—a feature noted in 1879 by Paul Ehrlich, who first described eosinophils and appreciated their increased presence in patients with asthma and helminth infections, among other conditions (4). See Figure 1 for an overview of the eosinophil ultrastructure.

The presence of large specific granules, also known as secondary granules, is a characteristic feature that distinguishes eosinophils from other granulocytes (neutrophils and basophils). Specific granules consist of a dense crystalline core and a matrix, surrounded by a membrane (5). They contain a large number of mediators capable of inducing inflammation and/or tissue damage, including basic proteins, cytokines, chemokines, growth factors, and enzymes. The predominant substances are the proteins: major basic protein (MBP) is located in the core, while the matrix contains eosinophil cationic protein (ECP), eosinophil peroxidase (EPO), and eosinophil-derived neurotoxin (EDN) (6).

Primary granules tend to be smaller than specific granules. They are the principal location of Charcot–Leyden crystal protein (galactin-10): hexagonal bipyramidal crystals, which exhibit lysophospholipase activity and have been identified in tissues subject to eosinophilic inflammation (7).

Lipid bodies are particularly important when considering the role of eosinophils in asthma, due to their involvement in the production of eicosanoids, including cysteinal leukotrienes, prostaglandins, and thromboxane (2). Lipid bodies are a key site of arachidonic acid esterification and eicosanoid production due to their high concentrations of relevant enzymes such as cyclooxygenases, 5-lipoxygenase, and leukotriene C4-synthase (5).

Golgi bodies, endoplasmic reticulum, and mitochondria are also present and fulfill the fundamental duties of protein and adenosine triphosphate production within the eosinophil.

The histological appearance of eosinophils varies depending on the level of activation. For example, higher numbers of vesicles such as eosinophil sombrero vesicles may be seen when the cell is undergoing piecemeal degranulation (PMD), a process described in detail further on.

## Eosinophil Surface Molecules and Receptors

The varied roles of the eosinophil are reflected in its wide repertoire of surface molecules and receptors, which integrate eosinophils with both the innate and adaptive immune systems.

### Cytokine and Growth Factor Receptors

The heterodimeric receptor for IL-5 is thought to be the most important cytokine receptor expressed by eosinophils. The beta-subunit is identical to the beta-subunit of the receptors for granulocyte–macrophage colony-stimulating factor (GM-CSF) and IL-3 (both of which are also present on eosinophil cell membranes). The alpha-subunit, IL-5Rα, is specific to IL-5 and has been identified as a therapeutic target for severe eosinophilic asthma and other eosinophil-mediated conditions. The IL-5 receptor is also expressed by basophils.

Eosinophils also express receptors for multiple other cytokines and growth factors, including for IL-4, IL-13, IL-33, thymic stromal lymphopoietin, and transforming growth factor-β (TGF-β).

### Chemoattractant Receptors

Chemokines are small cytokines, which stimulate the migration of specific subsets of leukocytes. Chemokines are divided into four groups, depending on the presence or absence of one or more interposing amino acid(s) between two cysteine residues (known as CXC-, CX3C-, and CC-chemokines), or the presence of only one cysteine residue (XC-chemokines) (8).

CC-chemokine receptor-3 (CCR3) is an important G protein-coupled receptor expressed on eosinophil cell membranes. CCR3 binds to all three subtypes of eotaxin (a variety of CC-chemokine that functions as a selective eosinophil chemoattractant). CCR3 also binds to several other chemokines including monocyte chemoattractant protein-3 (MCP-3) and MCP-4. The airways of patients with asthma have been shown to contain higher numbers of cells expressing mRNA for CCR3 and its ligands, compared to non-asthmatic control subjects (9). In mouse models of allergic airway inflammation, antigen-induced airway eosinophilia may be inhibited by the administration of either a monoclonal antibody against CCR3 (10) or a low molecular weight CCR3-antagonist (11).

CCR1 is another key chemokine receptor on eosinophils, which is activated by chemoattractant cytokine ligand-3 (CCL-3) and CCL-5 (also known as RANTES—regulated on activation, normal T cell expressed and secreted).

### Lipid Mediator Receptors

Eosinophils possess cell surface receptors for lipid mediators such as leukotrienes, prostaglandins, and platelet-activating factor, all of which have been shown to have a role in asthma pathophysiology (12–14).

### Pattern Recognition Receptors (PRRs)

Pattern recognition receptors react to microbial pathogen-associated molecular patterns (PAMPs) or host-derived damage-associated molecular patterns (DAMPs) and regulate the immune response to these indicators of infection and/or tissue damage (15).

Toll-like receptors (TLRs) are a family of PRRs, which are expressed by eosinophils, as well as multiple other cell lines. In humans there are 10 types of TLRs. TLRs are transmembrane glycoproteins, some of which are located at the cell surface and some in endosomes. The cytoplasmic domain resembles that of the IL-1 receptor, and the intracellular signals generated are therefore similar (16).

Eosinophils also express several other families of PRR, including retinoic acid-inducible gene-I-like receptors, nucleotide-binding oligomerisation domain-like (NOD-like) receptors, and the receptor for advanced glycation endproducts (RAGE) (15).

### Fc Receptors

Fc receptors to IgA, IgD, IgE, IgG, and IgM are expressed on the surface of eosinophils, facilitating interaction with the adaptive immune system.

The high-affinity FcεR1 receptor binds IgE and signals *via* intracellular tyrosine kinases. On mast cells and basophils, where FcεR1 is expressed as a tetramer (αβγ2), stimulation by IgE results in degranulation. However, on eosinophils, FcεR1 is usually expressed in very small quantities as a trimer (without a β chain) and has no role in eosinophil activation (17). In contrast, cross-linking of FcαRI and FcγRII, with IgA and IgG, respectively, has been shown to trigger eosinophil activation (18).

### Major Histocompatibility Complex-II (MHC-II)

Eosinophils have an additional role as antigen-presenting cells, facilitated by the presence of MHC-II molecules and co-stimulatory molecules such as CD80 and CD86. In allergic patients, evaluated after segmental antigen challenge, expression of HLA-DR (a subtype of MHC-II molecule) was found to be approximately four times greater in lung eosinophils compared to blood eosinophils (19).

### Adhesion Receptors

Adhesion receptors, as their name suggests, allow cells such as the eosinophil to adhere to the extracellular matrix (ECM) and to other cells. They also allow the eosinophil to sense its surroundings and respond accordingly. Adhesion receptors are divided into four main groups: integrins, cadherins, selectins, and immunoglobulin-like cell adhesion molecules (Ig-CAM). Integrins and selectins are the main forms of adhesion receptors expressed on eosinophil cell membranes.

Eosinophils express seven types of integrins, which are transmembrane glycoproteins, consisting of an α and a β chain (20). Examples include very late antigen-4 (VLA-4, CD49d/CD29) and the complement receptor CR3 (CD11b/CD18), which is otherwise known as macrophage-1 antigen (Mac-1).

Selectins are single-chain transmembrane glycoproteins with multiple domains. There are three families: E-, L-, and P-selectin; the latter two are expressed by human eosinophils, whereas E-selectin is present on activated endothelium (21).

### Siglec-8

Siglec-8 is a sialic acid immunoglobulin-like lectin (a carbohydrate-binding protein) expressed by eosinophils, mast cells, and basophils. Its physiological role has not yet been identified, although it is thought to represent a potential therapeutic target for eosinophil-mediated disease, due to the observation that administration of an antibody targeted against Siglec-8 can induce selective eosinophil apoptosis and inhibit mast cell degranulation (22).

## Eosinophil Differentiation, Maturation, Migration, Activation, and Degranulation

Eosinophils develop from pluripotent CD34+ granulocyte progenitor cells.

Differentiation and maturation occurs as follows:

Myeloblast→Promyelocyte→Eosinophil myelocyte→Eosinophil metamyelocyte→Eosinophil.

Allergen challenge of mild asthmatics results in increased expression of IL-5Rα on CD34+ cells in the bone marrow, associated with blood and sputum eosinophilia (23). Eosinophil differentiation usually occurs in the bone marrow. However, eosinophil precursors have been isolated from the peripheral blood of atopic subjects at significantly higher concentrations compared to non-atopic controls (24). Increased numbers of CD34+/IL-5Rα+ eosinophil precursors have also been identified in bronchial biopsies of atopic asthmatics, compared to non-asthmatic control subjects (both atopic and non-atopic) (25). Eosinophil-lineage committed cells have also been identified in lung tissue in a mouse model of allergic airway inflammation (26). More recently, eosinophil progenitors isolated from the blood of patients with severe eosinophilic asthma have been shown to have an exaggerated clonogenic response to IL-5 *in vitro*, compared to eosinophil precursors from mild asthmatics, suggesting that *in situ* eosinophilopoiesis may have a clinically relevant role in severe eosinophilic asthma (27).

The differentiation of eosinophils is regulated by the transcription factors GATA-binding protein 1 (GATA-1), PU.1, and the CCAAT-enhancing binding protein (c/EBP) family. GATA-1 and PU.1 synergistically promote transcription of MBP (28). GATA-1 is thought to have the most important role, as disruption of the GATA-1 gene in mice results in a strain completely devoid of eosinophils (29).

The cytokines IL-3, IL-5, and GM-CSF also synergistically contribute to the development of mature eosinophils (30). IL-5 is the most eosinophil-specific and also promotes the release of eosinophils from the bone marrow to the bloodstream, acting synergistically with eotaxin (31, 32). Eosinophils are present in relatively low numbers in peripheral blood, usually accounting for no more than 5% of the total white blood cell count (33). They have a relatively short blood half-life of approximately 18 h (34). Migration to specific body sites, including the lungs and intestines, is mediated by eosinophil chemoattractants such as eotaxins. Eotaxins are a variety of CC-chemokines. There are three known subtypes: eotaxin-1 (CCL-11), eotaxin-2 (CCL-24), and eotaxin-3 (CCL-26). These bind to CCR3 receptors on the cell membranes of eosinophils and induce chemotaxis. 5-oxo 6, 8, 11, 14-eicosatetraenoic acid (5-oxo-ETE) is another eosinophil chemoattractant.

*In vitro*, the presence of prostaglandin-D2 (PGD2) has been shown to significantly enhance the chemoattractant effects of eotaxin-1 and 5-oxo-ETE on eosinophils and—unlike eotaxin-1 or 5-oxo-ETE—PGD2 retains its chemoattractant effect in the presence of blood or plasma (35). It is therefore proposed that PGD2 acts as the initial chemoattractant, triggering the migration of circulating eosinophils to specific tissues, where eotaxins and 5-oxo-ETE then predominate. PGD2 is released from activated mast cells (36) and acts *via* CRTh2 (chemoattractant receptor-homologous molecule expressed on T_H_2 cells).

In allergic inflammation and asthma, circulating eosinophils adhere to the vascular endothelium and roll along it, before extravasating to the lung tissue. Initial tethering to the endothelium occurs as a result of the eosinophil cell membrane adhesion receptor P-selectin binding to P-selectin glycoprotein ligand-1 on the endothelium (37). Binding of the integrin VLA-4 to vascular cell adhesion molecule-1 promotes eosinophil activation and extravasation (37). IL-13 results in increased eosinophil expression of P-selectin and increased P-selectin mediated adhesion to endothelial cells (38).

The eosinophil’s ability to store several preformed cytotoxic mediators ready for rapid release upon appropriate stimulation facilitates a much quicker reaction to pro-inflammatory stimuli, compared to other cells, whose responses depend on upregulating the transcription of genes coding for such proteins.

The bronchial epithelium produces the cytokines IL-25, IL-33, and thymic stromal lymphopoietin, collectively known as the alarmins, in response to irritants such as allergens, pollutants, and pathogens. These cytokines trigger an inflammatory cascade involving, among others, T helper-2 (T_H_2) cells and type-2 innate lymphoid cells (ILC2s), resulting in increased production of numerous cytokines including IL-4, IL-5, and IL-13, therefore prompting eosinophil activation (1, 39).

High mobility group box 1 protein, acting *via* receptors TLR-2, TLR-4, and RAGE, also promotes eosinophilia, although less is known regarding its mechanism of action (2).

Specific granule contents may be released *via* three different degranulation processes. Conventional exocytosis entails the specific granules fusing with the eosinophil cell membrane, resulting in the release of the entire contents of the specific granule. Alternatively, the eosinophil may be lysed (cytolysis), releasing all the cell contents, including the intact specific granules. These extracellular granules can be found in tissues affected by eosinophil-mediated disease and may subsequently release their contents in response to pro-inflammatory stimuli (40). However, the most common mechanism of eosinophil granulation is termed piecemeal degranulation (PMD). In this process, vesicles (both round and tubular) are released from specific granules and travel to the cell membrane to discharge their contents to the extracellular domain (41). The tubular vesicles tend to curl into a hoop-like morphology, giving rise to the term “eosinophil sombrero vesicles” (42). Vesicles with particular contents may be selectively released in response to particular cytokines, allowing eosinophils to supply a specific combination of cytotoxic mediators on demand (42, 43).

The activation of TLRs on eosinophils has been shown to promote adhesion and the release of certain cytokines and superoxides (44). Activation of TLR-2 and TLR-9 triggers eosinophil degranulation (44, 45). *In vitro*, eosinophils from atopic subjects have been shown to produce more IL-8 and EDN in response to stimulation of TLR-7 and TLR-9, compared to healthy controls (45).

Eosinophil survival is promoted by IL-3, IL-5, GM-CSF, and eotaxin (37). Activation of TLR-7 (the most abundant TLR subtype expressed by eosinophils) also enhances eosinophil survival (45).

## IL-3, IL-5, and GM-CSF

Among the type-2 cytokines, IL-3, IL-5, and GM-CSF are particularly important for the initiation and perpetuation of eosinophilic airway inflammation. These three cytokines are closely linked, in that the genes for all three are all located on chromosome 5, and their receptors also share a common β-subunit (βc).

Monoclonal antibodies against IL-5 have been developed in order to treat eosinophil-mediated diseases such as eosinophilic asthma. Although inhibition of IL-5 activity in this manner (using mepolizumab) results in significant depletion of circulating eosinophils, the effect on bronchial tissue eosinophilia is less marked, with a median reduction of 55% (46). The residual tissue eosinophilia may reflect ongoing effects mediated by IL-3 and GM-CSF.

In a mouse model of allergic airways inflammation, allergen-induced lung tissue eosinophilia was abolished in mice bred to lack the common β-subunit, therefore incapable of responding to IL-3, IL-5, and GM-CSF (47). In the same study, lung tissue from βc-deficient mice was found to contain fewer myeloid dendritic cells, and the local T_H_2 cells showed a reduced ability to proliferate and produce type-2 cytokines (47). These findings suggest a multifactorial role for the common β-subunit in the regulation of allergic airway inflammation.

## The Role of the Eosinophil in Health

In comparison to the roles that eosinophils play in diseases and infections, relatively little is known about their purpose in health. However, an increasing number of homeostatic mechanisms have been attributed to—or at least associated with—eosinophils in recent years. This has prompted a call for a fundamental change of the perception of eosinophils purely as cytotoxic effector cells (48, 49).

In health, eosinophils are found in the thymus, spleen, lymph nodes, and gastrointestinal (GI) tract (50). The number of eosinophils in the thymus declines with age (51). Eosinophils may have a role in T cell selection. In a mouse model of MHC I-restricted acute negative selection, eosinophil recruitment to the corticomedullary region of the thymus and association with apoptotic bodies has been demonstrated (52). Eosinophils also enhance the ability of macrophages to phagocytose apoptotic thymic cells (53).

Eosinophils migrate to the GI tract during embryonic development, i.e., prior to the development of any viable gut flora (54). In health, they are present throughout the GI tract—with the notable exception of the esophagus. Eosinophils contribute to the immune defense against gut microorganisms, due to multiple antimicrobial properties. (The antimicrobial properties of eosinophils are discussed in detail further on, with specific relation to respiratory pathogens.) Other potential homeostatic roles for eosinophils within the gut are not currently well defined but may relate to their ability to interact with the enteric neuronal system and increase smooth muscle reactivity (*via* release of MBP) (55).

In murine white adipose tissue, a positive correlation was identified between eosinophil counts and the numbers of arginase-1-expressing macrophages (56). Macrophages expressing arginase-1 are thought to contribute to glucose homeostasis, although macrophage classification is contentious (57). In a more recent study involving more than 9,000 human participants, the peripheral blood eosinophil percentage was found to be inversely associated with the risk of type-2 diabetes mellitus and insulin resistance (58).

Eosinophils have also been implicated in the regeneration of liver tissue (59) and skeletal muscle (60). The increased presence of eosinophils in preovulatory ovarian follicles (61) and in endometrium (62) has prompted speculation that they may have a role in tissue remodeling related to ovulation and menstruation.

Eosinophils also perform several important immunomodulatory functions, discussed in the following section.

## The Eosinophil’s Role in Asthma Pathophysiology

**Figure 2:**
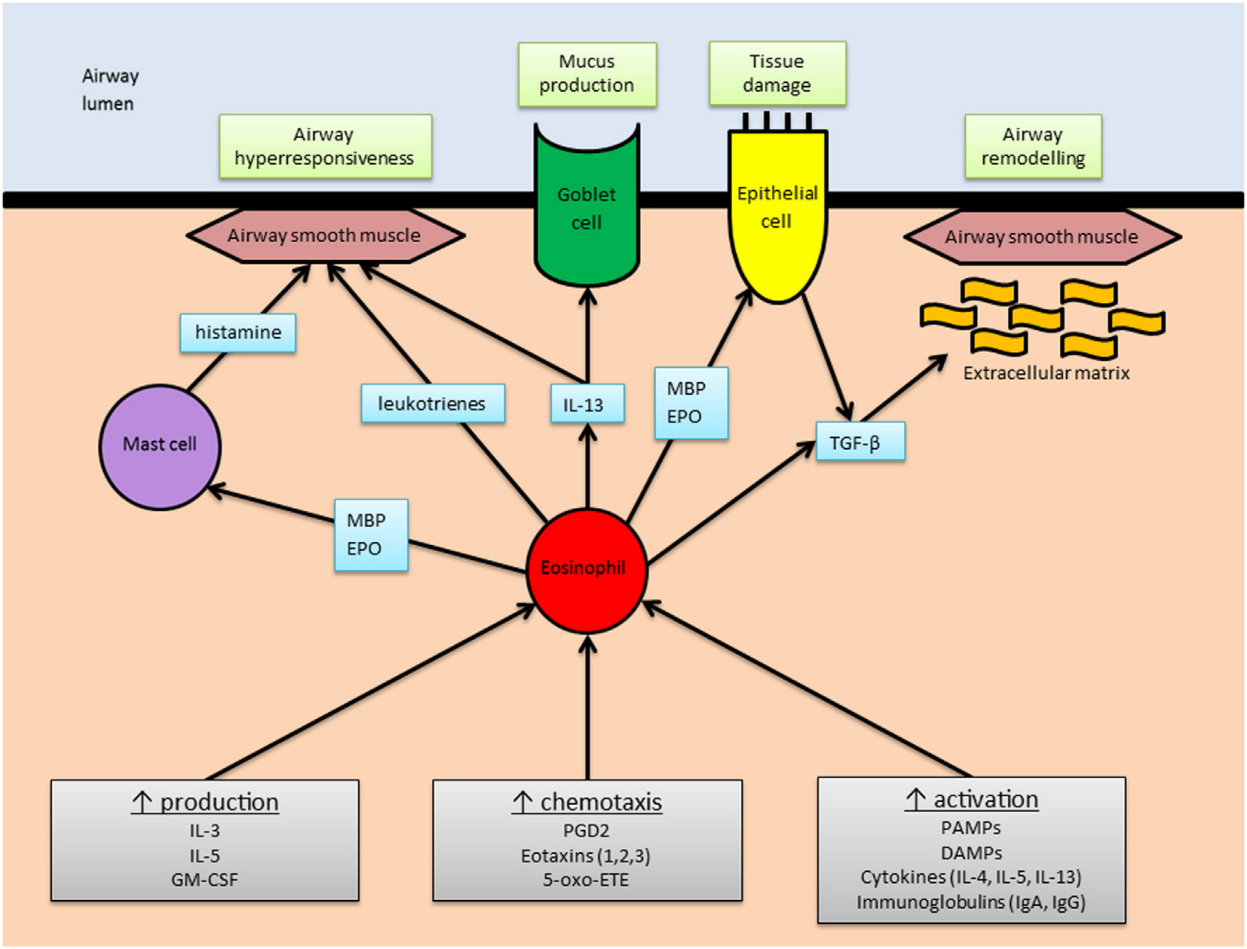
The role of eosinophils in asthma. An overview of the main stimuli for eosinophilic airway inflammation (gray boxes) and the means by which eosinophils elicit the main pathophysiological changes associated with asthma (green boxes). Abbreviations: MBP, major basic protein; EPO, eosinophil peroxidase; IL, interleukin; TGF-β, transforming growth factor-β; GM-CSF, granulocyte–macrophage colony-stimulating factor; PGD2, prostaglandin-D2; 5-oxo-ETE, 5-oxo 6, 8, 11, 14-eicosatetraenoic acid; PAMPs, pathogen associated molecular patterns; DAMPs, damage associated molecular patterns; Ig, immunoglobulin.

Asthma pathophysiology is complex, and the relative contributions of the various cytokine networks involved vary between patients. Core features include airway hyperresponsiveness (AHR), mucus hypersecretion, tissue damage, and airway remodeling. See Figure 2 for an overview of the eosinophil’s role in asthma pathophysiology.

It has long been observed that eosinophil counts in peripheral blood and bronchoalveolar lavage (BAL) fluid are higher in asthmatics compared to healthy controls (63). Analysis of BAL fluid obtained from patients with atopic asthma reveals increased expression of T_H_2 cytokines (64), including IL-5, which are strongly associated with eosinophilic inflammation (65). In general, the degree of eosinophilia correlates with disease severity and exacerbation frequency (63, 66). However, non-eosinophilic asthma phenotypes are also recognized (67). Peripheral blood eosinophilia may also occur in numerous other conditions (see Box 1).

Box 1 **Alternative (i.e., non-asthma) causes of peripheral eosinophilia (68, 69).**

Respiratory

 Eosinophilic granulomatosis with polyangiitis (EGPA)

 Allergic bronchopulmonary aspergillosis

 Sarcoidosis

Hematological and neoplastic

 Myeloproliferative hypereosinophilic syndrome

 Lymphocytic-variant hypereosinophilic syndrome

 Certain leukemias and lymphomas

 Systemic mastocytosis

 Solid tumors—adenocarcinomas, squamous cell carcinomas, large cell lung carcinomas, transitional cell carcinoma of the bladder

Infective

 Parasitic infection, in particular helminths

 Human immunodeficiency virus

Dermatological

 Eczema

 Scabies infestation

Iatrogenic

 Certain drug hypersensitivity reactions

 Graft vs host disease

### AHR and Mucus Hypersecretion

Eosinophils may be prompted to release a number of different mediators with the capacity to cause AHR. Human MBP is known to result in AHR when administered to primates (70) and rats (71). In the former study, administration of EPO also caused AHR, although ECP and EDN did not (70). Data from the latter study suggested the mechanism of action involved the stimulation of bradykinin production (71). MBP can also trigger mast cells and basophils to release histamine, a potent mediator of bronchial hyperreactivity (72, 73).

Eosinophils are a source of several cytokines including IL-13, which causes AHR, and also promotes mucus hypersecretion *via* enhanced differentiation of goblet cells (74). IL-13 is also produced by T_H_2 cells and ILC2s. Lipid mediators such as leukotrienes, which are produced in eosinophil lipid bodies (and mast cells), also cause AHR and mucus hypersecretion (75).

Studies involving two different strains of eosinophil-deficient mice have attempted to clarify the role of eosinophils in an ovalbumin model of asthma but yielded seemingly contradictory results. In one study, performed by Lee et al., eosinophil-deficient mice were protected from AHR and mucus hypersecretion (76). However, another study, led by Humbles, found that eosinophil deficiency was not protective in this regard (77). Several theories have been put forward to explain the conflicting results, including evidence of residual lung eosinophils in the Humbles study and differences between the underlying mouse strains (78).

In terms of practical application, the existence in humans of eosinophilic bronchitis, a condition characterized by marked airway eosinophilia in the absence of AHR, calls into question the concept that eosinophils—acting alone—have a clinically significant impact on AHR. In patients with mild asthma, administration of a monoclonal antibody to IL-5 has been shown to reduce blood and sputum eosinophilia but had no effect on AHR (79). This may reflect the cellular redundancy of AHR pathophysiology, which involves several cell types including T_H_2 cells, ILC2s, and mast cells. In addition, the current evidence relating to AHR does not specifically study the pathophysiology of asthma exacerbations, during which it is possible that eosinophil degranulation may contribute to worsening AHR.

### Tissue Damage and Airway Remodeling

Postulation that eosinophils are major effectors of lung tissue damage in asthma is well founded, given their propensity to release highly charged basic proteins, which exert multiple cytotoxic effects. MBP is toxic to respiratory epithelial cells *in vitro* and has been identified in postmortem lung tissue specimens of patients who have died of asthma, in association with epithelial damage (80). ECP and EDN share 67% amino acid sequence homology and tend to be grouped together as eosinophil-associated RNases, although ECP’s RNase activity is much less potent (81). ECP binds to cell membranes and alters their permeability (82). EDN, as its name suggests, is neurotoxic. It was first identified following the observation that, in rabbits, the intracerebral administration of eosinophils resulted in the destruction of cerebellar Purkinje cells and neurological features named “the Gordon phenomenon” (83, 84). EPO catalyzes the oxidation of halides and thiocyanate, resulting in cytotoxic reactive oxidant species (85).

Cell damage triggers the activation of repair pathways which, if excessive, may contribute to structural changes referred to as airway remodeling. The underlying pathological processes include hyperplasia of fibroblasts, airway smooth muscle (ASM) and goblet cells, deposition of ECM proteins, and angiogenesis (86). Airway remodeling is associated with the severity of asthma (87). It has been hypothesized that airway remodeling is responsible for the accelerated decline in lung function and development of fixed airway obstruction observed in some asthmatic patients. However, bronchial biopsies of children with difficult asthma have been shown to display reticular basement membrane thickening to a similar degree compared with adult asthmatics (88). Furthermore, there is evidence that some pathological features of airway remodeling can become evident within 24 h of allergen exposure (89).

Eosinophils release multiple growth factors and fibrogenic mediators that promote airway remodeling (see Table 1). For example, eosinophils are known to produce TGF-β in disease states involving the skin (atopy) (90), nose (nasal polyposis) (91), and blood (idiopathic hypereosinophilic syndrome) (92). Eosinophils are the main source of TGF-β in bronchial biopsies taken from asthmatic patients (93) and can also stimulate epithelial cells to produce a number of mediators, including TGF-β (94). TGF-β is implicated in tissue remodeling *via* fibroblast proliferation and increased production of collagen and glycosaminoglycans (95–97).

**Table 1:** Factors produced by eosinophils that are associated with airway remodeling.

Factor	Mechanism(s) and evidence
TGF-β	Epithelial/submucosal expression of TGF-β correlates with basement membrane thickness and fibroblast numbers (98). In allergen-challenged human atopic skin, eosinophils expressing TGF-β1 are associated with myofibroblast formation and deposition of tenascin and procollagen-1 (90). TGF-β induces hypertrophy and increased contractility of ASM *in vitro* (99). Administration of anti-TGF-β antibody to mice with established eosinophilic airway inflammation significantly reduces airway remodelling (100).
MMP-9 and TIMP-1	MMP-9 breaks down ECM proteins; TIMP-1 inhibits MMP-9. Sputum MMP-9 and TIMP-1 concentrations are higher in asthmatics compared to controls; The MMP-9/TIMP-1 ratio is lower in patients with asthma and chronic bronchitis, and positively correlates with FEV1 (101). MMP-9 is required for angiogenesis in mice (102).
VEGF, bFGF, and angiogenin	VEGF, bFGF, and angiogenin promote angiogenesis. Bronchial biopsies of asthmatics exhibit greater immunoreactivity to VEGF, bFGF, and angiogenin; Immunoreactivity to these factors positively correlates with vascular area (103).
Specific granule proteins	MBP and ECP are toxic to airway epithelial cells. Damaged airway epithelium produces TGF-β (104). ECP induces fibroblast migration (105) and inhibits fibroblast-mediated proteoglycan degradation (106).
IL-17	Fibroblasts isolated from bronchial biopsies produce more IL-6 and IL-11 (profibrotic cytokines) when stimulated by IL-17 (107). In a mouse model of asthma, administration of IL-17A results in increased vascular remodelling; *in vitro*, IL-17A accelerates EPC migration (108).
IL-13	Mice bred to overexpress IL-13 exhibit eosinophilic airway inflammation, epithelial cell hypertrophy, mucus metaplasia, and subepithelial fibrosis (109). *In vitro*, IL-13 induces human bronchial epithelial cells to release TGF-β (110).
HB-EGF	Recombinant HB-EGF promotes migration of ASM cells *in vitro* and accelerates the thickening of the ASM layer in a mouse model of asthma (111).
NGF	NGF causes migration of vascular smooth muscle cells and fibroblasts, and proliferation of epithelial cells and ASM cells (112). In mice with chronic allergen-induced airway inflammation, anti-NGF antibodies reduce airway collagen deposition (113).
Cysteinyl leukotrienes	In a mouse model of allergen-induced airway remodelling, administration of montelukast (a CysLT1 receptor blocker) reverses established ASM layer thickening and subepithelial fibrosis (114).
SCF	SCF promotes mast cell proliferation and activation. Mast cells produce TNF-α, which can damage bronchial epithelial cells (115) and stimulate fibroblasts to produce TGF-β (116).

*TGF, transforming growth factor; MMP, matrix metalloproteinase; ECM, extracellular matrix; TIMP, tissue inhibitor of metalloproteinase; FEV1, forced expiratory volume in 1 s; VEGF, vascular endothelial growth factor; bFGF, basic fibroblast growth factor; MBP, major basic protein; ECP, eosinophil cationic protein; IL, interleukin; EPC, endothelial progenitor cell; HB-EGF, heparin-binding epidermal growth factor-like growth factor; ASM, airway smooth muscle; NGF, nerve growth factor; CysLT1, cysteinyl leukotriene 1; SCF, stem cell factor; TNF, tumor necrosis factor*.

Eosinophils isolated from asthmatics, when cocultured with ASM cells, promote enhanced ASM proliferation, which is inhibited by the addition of the leukotriene antagonist montelukast (117). It appears that eosinophils and ASM enjoy a reciprocal relationship, as ASM cells are also known to produce pro-eosinophil cytokines (118). Mouse studies lend further support to the assertion that eosinophils have an important role in airway remodeling, as eosinophil-deficient mice are protected against airway deposition of collagen and smooth muscle (77).

Treatment with the anti-IL-5 monoclonal antibody mepolizumab has been shown to reduce bronchial tissue eosinophilia, in association with decreased TGF-β1 in BAL specimens, and reduced reticular basement membrane procollagen III, tenascin, and lumican (119).

### Asthma Exacerbations

Airway eosinophilia is an early feature of asthma exacerbations. In a study of steroid-dependent asthmatic patients, whose prednisolone dose was gradually reduced to below their maintenance requirement, the sputum eosinophil count started to rise 4 weeks before the blood eosinophil count and 6 weeks prior to spirometric and symptomatic deterioration (120). In fact, the adoption of an asthma treatment strategy based on sputum eosinophilia rather than traditional markers of disease activity (such as symptoms and spirometry) was found to reduce the frequency of exacerbations, with no overall increase in the average daily corticosteroid dose (121).

The primary action of anti-IL-5 therapies appears to be a reduction in exacerbation frequency. Administration of mepolizumab to selected patient groups reduced exacerbation rates by approximately 50% (122–124). A similar reduction in exacerbation rates was seen with reslizumab, another anti-IL-5 monoclonal antibody (125). Mepolizumab has also been found to have a moderate glucocorticoid-sparing effect in a phase III clinical trial (126).

Benralizumab is a monoclonal antibody targeted against the alpha subunit of the IL-5 receptor (IL-5Rα). As well as blocking the interaction between IL-5 and its receptor, benralizumab causes eosinophil cell death *via* antibody-dependent cell-mediated cytotoxicity (127), resulting in striking (95%) airway eosinophil depletion (128). Phase III clinical trials have demonstrated reductions in exacerbation rates (129, 130).

### Immunomodulation

In addition to the direct effects of eosinophils on asthma pathophysiology, they have an important role in immunomodulation (2). MBP, released from eosinophil-specific granules, stimulates inflammatory responses from neutrophils (increased production of superoxide and IL-8) (131) and mast cells (increased histamine release) (72). Nerve growth factor (also released from specific granules) has also been shown to prolong the survival of neutrophils (132) and mast cells (133). EDN promotes the activation of dendritic cells (134), which in turn trigger the proliferation of T cells (both helper and cytotoxic) and B cells *via* antigen presentation. Eosinophils themselves can also present antigens to T cells (135, 136).

The cytokines released from eosinophil-specific granules have various immunomodulatory effects. For example, IL-4 and IL-13 simulate the proliferation of B cells and IgE production (137, 138), and IL-6 enhances survival of plasma cells (139, 140) Eosinophil-specific granules are also capable of releasing several chemokines. Depending on the stimulation they receive, these include CCL-17 and CCL-22, which attract T_H_2 cells, and CXCL-9 and CXCL-10, which are T_H_1 chemokines (141). In addition, eosinophils express indoleamine 2,3-dioxygenase (IDO), an enzyme that catalyzes the production of kynurenine, suppressing T_H_1 activity and promoting a type-2 inflammatory milieu (142, 143).

**Figure 3:**
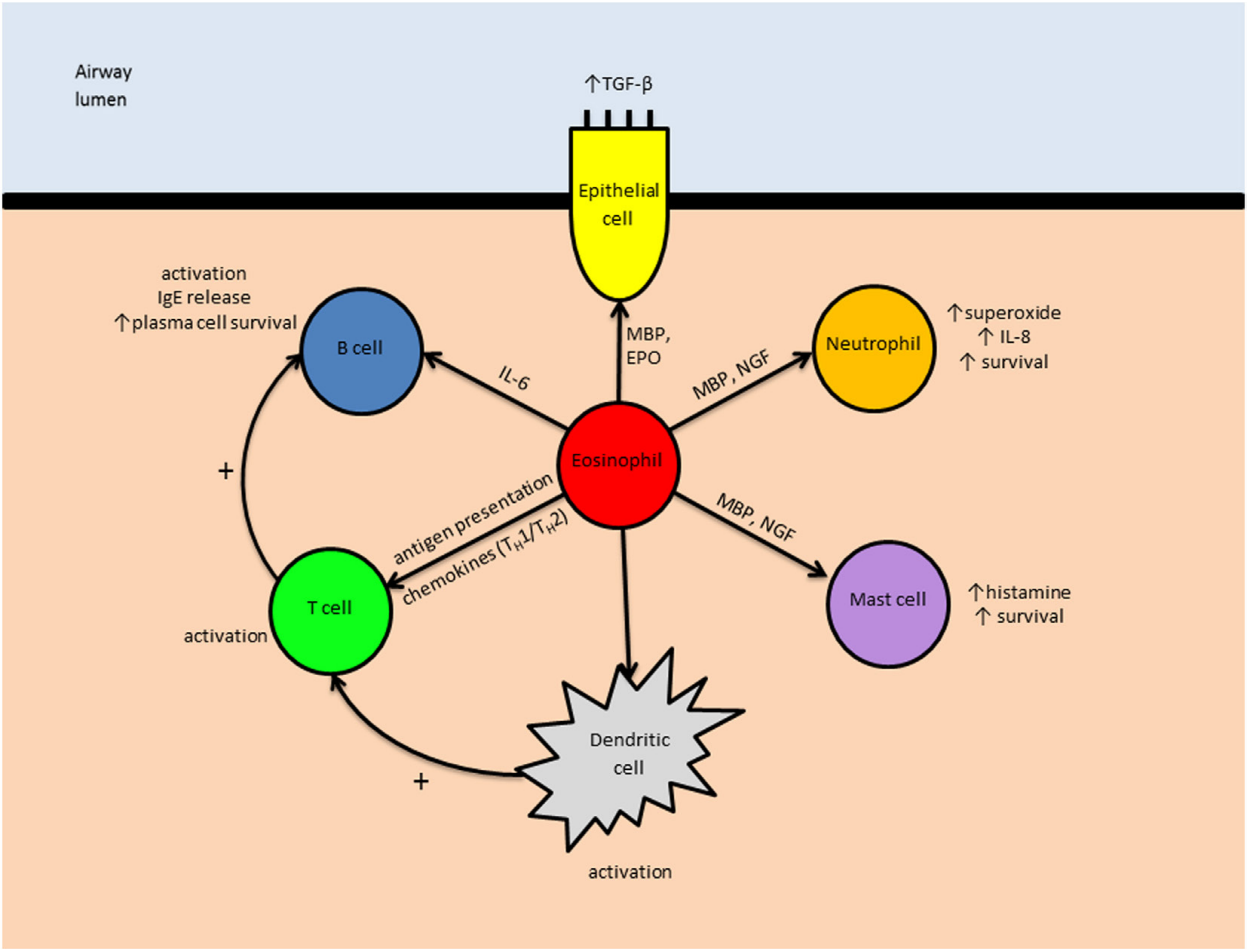
The immunomodulatory role of eosinophils in asthma. Eosinophils may influence other leukocytes both directly (e.g., IL-6-induced B cell activation) and indirectly (e.g., by enhancing antigen presentation by dendritic cells). Abbreviations: TGF-β, transforming growth factor-β; IgE, immunoglobulin E; IL, interleukin; MBP, major basic protein; EPO, eosinophil peroxidase; NGF, nerve growth factor; T_H_1, type 1 T helper cell; T_H_2, type 2 T helper cell.

See Figure 3 for an overview of the eosinophil’s immunomodulatory roles in asthma.

## Interactions Between Eosinophils and Respiratory Pathogens

Eosinophils have traditionally been regarded as end-stage effector cells, responding to infections directly, i.e., by releasing substances that are toxic to pathogens (in particular, helminths) and resulting in the unwanted secondary effect of human tissue damage. However, research performed over the last 30 years has revealed additional roles fulfilled by the eosinophil, involving links with both the innate and adaptive immune systems. These roles include antigen presentation and interaction with other parts of the immune system, such as the complement pathway (144).

Pattern recognition receptors on the cell membranes of eosinophils allow them to detect the presence of PAMPs such as lipopolysaccharide (LPS) and beta-glucans, cell wall components of bacteria and fungi, respectively (144). The cysteine and serine proteases produced by mites and fungi activate eosinophils *via* protease-activated receptors (5). TLR-7, the most common TLR expressed by eosinophils, is activated by viral single-stranded RNA (2).

The contents of eosinophil-specific granules are directly cytotoxic to pathogens. MBP causes disruption of cell membranes due to its highly basic nature (145). ECP has antiviral activity (146) and can also agglutinate Gram-negative bacteria by binding to LPS and peptidoglycans (147). EDN is only mildly toxic to helminths, compared to MBP and ECP (148). However, EDN significantly reduces the infectivity of respiratory syncytial virus group B, indicating a role in the immune response to viruses (149). EPO facilitates the generation of toxic reactive oxygen species (150).

In addition to releasing cytotoxic proteins, eosinophils have been shown to phagocytose bacteria (albeit less efficiently than neutrophils) (151). More recently, the “catapult-like” extrusion of “traps” consisting of mitochondrial DNA and eosinophil granule contents, in response to Gram-negative bacteria, has been observed (152).

Eosinophils express receptors for various complement proteins, including C3a and C5a, which are known to promote eosinophil recruitment, extravasation, and activation (153, 154). Complement is thought to facilitate eosinophil adherence to, and damage of, nematode larvae (155), although the development of secondary immunity is unaffected in complement-deficient mice (156).

As fungi are known to trigger the production of T_H_2-associated cytokines (i.e., type-2 cytokines) and eosinophilia, it has been hypothesized that subclinical fungal infection/colonization of the airways may play a role in the genesis of diseases characterized by eosinophilia. Such diseases include severe eosinophilic asthma, as well as related conditions (e.g., chronic rhinosinusitis). One study of patients undergoing sinus surgery found that 74% of those with T_H_2-associated conditions had evidence of airway surface mycosis, compared to just 16% of controls (157). However, potential confounding factors such as inhaled and/or systemic corticosteroid usage must be considered.

The increased susceptibility to respiratory viral infections observed in patients with asthma has been linked to reduced production of type I and type III interferons (158, 159). Eosinophils may contribute to this impairment by producing TGF-β, which has been shown to diminish the ability of bronchial epithelial cells to produce interferons in response to human rhinovirus *in vitro* (160).

The lung is known to harbor communities of bacteria, known as the lung microbiome, during health, which are deranged in disease states including asthma (161, 162). Data have recently been published suggesting a possible link between the level of eosinophilia and microbiome community structure in asthma (163). Further dedicated studies, examining subject groups matched for baseline characteristics, are required.

## Conclusion

Although eosinophils have been associated with asthma since their initial discovery, our understanding of their roles in health and disease has evolved significantly over time. The eosinophil’s status as a cytotoxic effector cell appears to be justified, due its capacity to release potent destructive basic proteins, capable of antimicrobial effects as well as host tissue damage. However, its ability to modulate the innate and adaptive immune systems may be just as important.

An appreciation of the numerous receptors expressed by eosinophils offers some insight into the many different interactions this versatile cell is capable of. Not only is the eosinophil recruited to the lungs in the context of pro-inflammatory type-2 cytokines but it is also a promoter of the type-2 inflammatory milieu, taking on roles such as antigen presentation and cytokine-mediated modulation of local lymphocytes.

There is strong evidence that eosinophils contribute to airway remodeling in asthma. Mechanisms also exist by which eosinophils could promote AHR and mucus hypersecretion.

The development of new anti-eosinophilic drugs, capable of selective depletion of eosinophils, offers great potential to explore further questions relating to the role of eosinophils in asthma and the consequences of their eradication. Research into variation in eosinophil-related gene expression between individuals may provide further insights regarding the relative contributions of eosinophils in different asthma phenotypes and the potential application of personalized medicine to this field.

## Author Contributions

CM performed the initial literature review and drafted the article. AM-G provided critical review and additional content.

## Conflict of Interest Statement

CM has attended an international conference with Boehringer Ingelheim. AMG has attended advisory boards for Glaxo SmithKline, Novartis, Astra Zeneca, Boehringer Ingelheim and Teva. He has received speaker fees from Novartis, Astra Zeneca, Vectura, Boehringer Ingelheim and Teva. He has participated in research for which his institution has been renumerated with Hoffman La Roche, Glaxo SmithKline and Boehringer Ingelheim. He has attended international conferences with Napp and Astra Zeneca and has consultancy agreements with Astra Zeneca and Vectura.

## References

[1] Lambrecht BN Hammad H The immunology of asthma. Nat Immunol (2015) 16(1):45–56. 10.1038/ni.3049 25521684

[2] Rosenberg HF Dyer KD Foster PS. Eosinophils: changing perspectives in health and disease. Nat Rev Immunol (2013) 13(1):9–22. 10.1038/nri3341 23154224 PMC4357492

[3] Dahl R. Systemic side effects of inhaled corticosteroids in patients with asthma. Respir Med (2006) 100(8):1307–17. 10.1016/j.rmed.2005.11.020 16412623

[4] Gleich GJ Chapter 1 – Historical overview and perspective on the role of the eosinophil in health and disease A2 – Lee, James J. In: Rosenberg HF, editor. Eosinophils in Health and Disease. Boston: Academic Press (2013). p. 1–11.

[5] Shamri R Xenakis JJ Spencer LA. Eosinophils in innate immunity: an evolving story. Cell Tissue Res (2011) 343(1):57–83. 10.1007/s00441-010-1049-6 21042920 PMC3679536

[6] Giembycz MA Lindsay MA Pharmacology of the eosinophil. Pharmacol Rev (1999) 51(2):213–340. 10353986

[7] Dvorak AM Letourneau L Login GR Weller PF Ackerman SJ. Ultrastructural localization of the Charcot-Leyden crystal protein (lysophospholipase) to a distinct crystalloid-free granule population in mature human eosinophils. Blood (1988) 72(1):150–8. 2455566

[8] Rossi D Zlotnik A. The biology of chemokines and their receptors. Annu Rev Immunol (2000) 18:217–42. 10.1146/annurev.immunol.18.1.217 10837058

[9] Ying S Meng Q Zeibecoglou K Robinson DS Macfarlane A Humbert M Eosinophil chemotactic chemokines (eotaxin, eotaxin-2, RANTES, monocyte chemoattractant protein-3 (MCP-3), and MCP-4), and C-C chemokine receptor 3 expression in bronchial biopsies from atopic and nonatopic (intrinsic) asthmatics. J Immunol (1999) 163(11):6321–9. 10570327

[10] Justice JP Borchers MT Crosby JR Hines EM Shen HH Ochkur SI Ablation of eosinophils leads to a reduction of allergen-induced pulmonary pathology. Am J Physiol Lung Cell Mol Physiol (2003) 284(1):L169–78. 10.1152/ajplung.00260.2002 12388345

[11] Komai M Tanaka H Nagao K Ishizaki M Kajiwara D Miura T A novel CC-chemokine receptor 3 antagonist, Ki19003, inhibits airway eosinophilia and subepithelial/peribronchial fibrosis induced by repeated antigen challenge in mice. J Pharmacol Sci (2010) 112(2):203–13. 10.1254/jphs.09277FP 20134116

[12] Singh RK Tandon R Dastidar SG Ray A. A review on leukotrienes and their receptors with reference to asthma. J Asthma (2013) 50(9):922–31. 10.3109/02770903.2013.823447 23859232

[13] Bisgaard H. Leukotrienes and prostaglandins in asthma. Allergy (1984) 39(6):413–20. 10.1111/j.1398-9995.1984.tb01963.x 6208802

[14] Palgan K Bartuzi Z. Platelet activating factor in allergies. Int J Immunopathol Pharmacol (2015) 28(4):584–9. 10.1177/0394632015600598 26486136

[15] Takeuchi O Akira S Pattern recognition receptors and inflammation. Cell (2010) 140(6):805–20. 10.1016/j.cell.2010.01.022 20303872

[16] Dembic Z The Function of Toll-Like Receptors, in Madame Curie Bioscience Database [Internet]. Austin, TX: Landes Bioscience (2000–2013).

[17] Stone KD Prussin C Metcalfe DD. IgE, mast cells, basophils, and eosinophils. J Allergy Clin Immunol (2010) 125(2 Suppl 2):S73–80. 10.1016/j.jaci.2009.11.017 20176269 PMC2847274

[18] Muraki M Gleich GJ Kita H. Antigen-specific IgG and IgA, but not IgE, activate the effector functions of eosinophils in the presence of antigen. Int Arch Allergy Immunol (2011) 154(2):119–27. 10.1159/000320226 20733320

[19] Sedgwick JB Calhoun WJ Vrtis RF Bates ME McAllister PK Busse WW. Comparison of airway and blood eosinophil function after in vivo antigen challenge. J Immunol (1992) 149(11):3710–8. 1358975

[20] Johansson MW Kelly EA Busse WW Jarjour NN Mosher DF. Up-regulation and activation of eosinophil integrins in blood and airway after segmental lung antigen challenge. J Immunol (2008) 180(11):7622–35. 10.4049/jimmunol.180.11.7622 18490765 PMC2585992

[21] Michail S Mezoff E Abernathy F. Role of selectins in the intestinal epithelial migration of eosinophils. Pediatr Res (2005) 58(4):644–7. 10.1203/01.PDR.0000180572.65751.F4 16189187

[22] Kiwamoto T Kawasaki N Paulson JC Bochner BS. Siglec-8 as a drugable target to treat eosinophil and mast cell-associated conditions. Pharmacol Ther (2012) 135(3):327–36. 10.1016/j.pharmthera.2012.06.005 22749793 PMC3587973

[23] Sehmi R Wood LJ Watson R Foley R Hamid Q O’Byrne PM Allergen-induced increases in IL-5 receptor alpha-subunit expression on bone marrow-derived CD34+ cells from asthmatic subjects. A novel marker of progenitor cell commitment towards eosinophilic differentiation. J Clin Invest (1997) 100(10):2466–75. 10.1172/JCI119789 9366561 PMC508447

[24] Sehmi R Howie K Sutherland DR Schragge W O’Byrne PM Denburg JA. Increased levels of CD34+ hemopoietic progenitor cells in atopic subjects. Am J Respir Cell Mol Biol (1996) 15(5):645–55. 10.1165/ajrcmb.15.5.8918371 8918371

[25] Robinson DS Damia R Zeibecoglou K Molet S North J Yamada T CD34(+)/interleukin-5Ralpha messenger RNA+ cells in the bronchial mucosa in asthma: potential airway eosinophil progenitors. Am J Respir Cell Mol Biol (1999) 20(1):9–13. 10.1165/ajrcmb.20.1.3449 9870912

[26] Southam DS Widmer N Ellis R Hirota JA Inman MD Sehmi R. Increased eosinophil-lineage committed progenitors in the lung of allergen-challenged mice. J Allergy Clin Immunol (2005) 115(1):95–102. 10.1016/j.jaci.2004.09.022 15637553

[27] Sehmi R Smith SG Kjarsgaard M Radford K Boulet LP Lemiere C Role of local eosinophilopoietic processes in the development of airway eosinophilia in prednisone-dependent severe asthma. Clin Exp Allergy (2016) 46(6):793–802. 10.1111/cea.12695 26685004

[28] Du J Stankiewicz MJ Liu Y Xi Q Schmitz JE Lekstrom-Himes JA Novel combinatorial interactions of GATA-1, PU.1, and C/EBPepsilon isoforms regulate transcription of the gene encoding eosinophil granule major basic protein. J Biol Chem (2002) 277(45):43481–94. 10.1074/jbc.M204777200 12202480

[29] Yu C Cantor AB Yang H Browne C Wells RA Fujiwara Y Targeted deletion of a high-affinity GATA-binding site in the GATA-1 promoter leads to selective loss of the eosinophil lineage in vivo. J Exp Med (2002) 195(11):1387–95. 10.1084/jem.20020656 12045237 PMC2193547

[30] Blanchard C Rothenberg ME. Biology of the eosinophil. Adv Immunol (2009) 101:81–121. 10.1016/S0065-2776(08)01003-1 19231593 PMC4109275

[31] Faccioli LH Mokwa VF Silva CL Rocha GM Araujo JI Nahori MA IL-5 drives eosinophils from bone marrow to blood and tissues in a guinea-pig model of visceral larva migrans syndrome. Mediators Inflamm (1996) 5(1):24–31. 10.1155/S096293519600004X 18475693 PMC2365769

[32] Palframan RT Collins PD Williams TJ Rankin SM. Eotaxin induces a rapid release of eosinophils and their progenitors from the bone marrow. Blood (1998) 91(7):2240–8. 9516121

[33] Blumenreich MS The white blood cell and differential count. 3rd ed In: Walker HW Hurst JW, editors. Clinical Methods: The History, Physical, and Laboratory Examinations. Boston: Butterworths (1990). Available from: https://www.ncbi.nlm.nih.gov/books/NBK261/#A4534 21250045

[34] Steinbach KH Schick P Trepel F Raffler H Döhrmann J Heilgeist G Estimation of kinetic parameters of neutrophilic, eosinophilic, and basophilic granulocytes in human blood. Blut (1979) 39(1):27–38. 10.1007/BF01008072 223692

[35] Schratl P Sturm EM Royer JF Sturm GJ Lippe IT Peskar BA Hierarchy of eosinophil chemoattractants: role of p38 mitogen-activated protein kinase. Eur J Immunol (2006) 36(9):2401–9. 10.1002/eji.200535672 16906532

[36] Lewis RA Soter NA Diamond PT Austen KF Oates JA Roberts LJ II. Prostaglandin D2 generation after activation of rat and human mast cells with anti-IgE. J Immunol (1982) 129(4):1627–31. 6809826

[37] Rosenberg HF Phipps S Foster PS. Eosinophil trafficking in allergy and asthma. J Allergy Clin Immunol (2007) 119(6):1303–10; quiz 1311–2. 10.1016/j.jaci.2007.03.048 17481712

[38] Woltmann G McNulty CA Dewson G Symon FA Wardlaw AJ. Interleukin-13 induces PSGL-1/P-selectin-dependent adhesion of eosinophils, but not neutrophils, to human umbilical vein endothelial cells under flow. Blood (2000) 95(10):3146–52. 10807781

[39] Fallon PG Ballantyne SJ Mangan NE Barlow JL Dasvarma A Hewett DR Identification of an interleukin (IL)-25-dependent cell population that provides IL-4, IL-5, and IL-13 at the onset of helminth expulsion. J Exp Med (2006) 203(4):1105–16. 10.1084/jem.20051615 16606668 PMC2118283

[40] Neves JS Perez SA Spencer LA Melo RC Reynolds L Ghiran I Eosinophil granules function extracellularly as receptor-mediated secretory organelles. Proc Natl Acad Sci U S A (2008) 105(47):18478–83. 10.1073/pnas.0804547105 19017810 PMC2587599

[41] Muniz VS Weller PF Neves JS Eosinophil crystalloid granules: structure, function, and beyond. J Leukoc Biol (2012) 92(2):281–8. 10.1189/jlb.0212067 22672875 PMC3395420

[42] Melo RC Spencer LA Perez SA Ghiran I Dvorak AM Weller PF. Human eosinophils secrete preformed, granule-stored interleukin-4 through distinct vesicular compartments. Traffic (2005) 6(11):1047–57. 10.1111/j.1600-0854.2005.00344.x 16190985 PMC2715427

[43] Lacy P Mahmudi-Azer S Bablitz B Hagen SC Velazquez JR Man SF Rapid mobilization of intracellularly stored RANTES in response to interferon-gamma in human eosinophils. Blood (1999) 94(1):23–32. 10381494

[44] Wong CK Cheung PF Ip WK Lam CW. Intracellular signaling mechanisms regulating toll-like receptor-mediated activation of eosinophils. Am J Respir Cell Mol Biol (2007) 37(1):85–96. 10.1165/rcmb.2006-0457OC 17332440

[45] Mansson A Cardell LO. Role of atopic status in toll-like receptor (TLR)7- and TLR9-mediated activation of human eosinophils. J Leukoc Biol (2009) 85(4):719–27. 10.1189/jlb.0808494 19129482

[46] Flood-Page PT Menzies-Gow AN Kay AB Robinson DS. Eosinophil’s role remains uncertain as anti-interleukin-5 only partially depletes numbers in asthmatic airway. Am J Respir Crit Care Med (2003) 167(2):199–204. 10.1164/rccm.200208-789OC 12406833

[47] Asquith KL Ramshaw HS Hansbro PM Beagley KW Lopez AF Foster PS. The IL-3/IL-5/GM-CSF common receptor plays a pivotal role in the regulation of Th2 immunity and allergic airway inflammation. J Immunol (2008) 180(2):1199–206. 10.4049/jimmunol.180.2.1199 18178860

[48] Lee JJ Jacobsen EA McGarry MP Schleimer RP Lee NA. Eosinophils in health and disease: the LIAR hypothesis. Clin Exp Allergy (2010) 40(4):563–75. 10.1111/j.1365-2222.2010.03484.x 20447076 PMC2951476

[49] Jacobsen EA Helmers RA Lee JJ Lee NA. The expanding role(s) of eosinophils in health and disease. Blood (2012) 120(19):3882–90. 10.1182/blood-2012-06-330845 22936660 PMC3496950

[50] Kato M Kephart GM Talley NJ Wagner JM Sarr MG Bonno M Eosinophil infiltration and degranulation in normal human tissue. Anat Rec (1998) 252(3):418–25. 10.1002/(SICI)1097-0185(199811)252:3<418::AID-AR10>3.0.CO;2-1 9811220

[51] Tulic MK Sly PD Andrews D Crook M Davoine F Odemuyiwa SO Thymic indoleamine 2,3-dioxygenase-positive eosinophils in young children: potential role in maturation of the naive immune system. Am J Pathol (2009) 175(5):2043–52. 10.2353/ajpath.2009.090015 19815714 PMC2774068

[52] Throsby M Herbelin A Pléau JM Dardenne M. CD11c+ eosinophils in the murine thymus: developmental regulation and recruitment upon MHC class I-restricted thymocyte deletion. J Immunol (2000) 165(4):1965–75. 10.4049/jimmunol.165.4.1965 10925279

[53] Kim HJ Alonzo ES Dorothee G Pollard JW Sant’Angelo DB. Selective depletion of eosinophils or neutrophils in mice impacts the efficiency of apoptotic cell clearance in the thymus. PLoS One (2010) 5(7):e11439. 10.1371/journal.pone.0011439 20625428 PMC2897847

[54] Mishra A Hogan SP Lee JJ Foster PS Rothenberg ME. Fundamental signals that regulate eosinophil homing to the gastrointestinal tract. J Clin Invest (1999) 103(12):1719–27. 10.1172/JCI6560 10377178 PMC408388

[55] Jacoby DB Gleich GJ Fryer AD. Human eosinophil major basic protein is an endogenous allosteric antagonist at the inhibitory muscarinic M2 receptor. J Clin Invest (1993) 91(4):1314–8. 10.1172/JCI116331 8473484 PMC288101

[56] Wu D Molofsky AB Liang HE Ricardo-Gonzalez RR Jouihan HA Bando JK Eosinophils sustain adipose alternatively activated macrophages associated with glucose homeostasis. Science (2011) 332(6026):243–7. 10.1126/science.1201475 21436399 PMC3144160

[57] Murray PJ Allen JE Biswas SK Fisher EA Gilroy DW Goerdt S Macrophage activation and polarization: nomenclature and experimental guidelines. Immunity (2014) 41(1):14–20. 10.1016/j.immuni.2014.06.008 25035950 PMC4123412

[58] Zhu L Su T Xu M Xu Y Li M Wang T Eosinophil inversely associates with type 2 diabetes and insulin resistance in Chinese adults. PLoS One (2013) 8(7):e67613. 10.1371/journal.pone.0067613 23894289 PMC3718808

[59] Goh YP Henderson NC Heredia JE Red Eagle A Odegaard JI Lehwald N Eosinophils secrete IL-4 to facilitate liver regeneration. Proc Natl Acad Sci U S A (2013) 110(24):9914–9. 10.1073/pnas.1304046110 23716700 PMC3683773

[60] Heredia JE Mukundan L Chen FM Mueller AA Deo RC Locksley RM Type 2 innate signals stimulate fibro/adipogenic progenitors to facilitate muscle regeneration. Cell (2013) 153(2):376–88. 10.1016/j.cell.2013.02.053 23582327 PMC3663598

[61] Aust G Simchen C Heider U Hmeidan FA Blumenauer V Spanel-Borowski K. Eosinophils in the human corpus luteum: the role of RANTES and eotaxin in eosinophil attraction into periovulatory structures. Mol Hum Reprod (2000) 6(12):1085–91. 10.1093/molehr/6.12.1085 11101691

[62] Jeziorska M Salamonsen LA Woolley DE. Mast cell and eosinophil distribution and activation in human endometrium throughout the menstrual cycle. Biol Reprod (1995) 53(2):312–20. 10.1095/biolreprod53.2.312 7492683

[63] Bousquet J Chanez P Lacoste JY Barnéon G Ghavanian N Enander I Eosinophilic inflammation in asthma. N Engl J Med (1990) 323(15):1033–9. 10.1056/NEJM199010113231505 2215562

[64] Robinson DS Hamid Q Ying S Tsicopoulos A Barkans J Bentley AM Predominant TH2-like bronchoalveolar T-lymphocyte population in atopic asthma. N Engl J Med (1992) 326(5):298–304. 10.1056/NEJM199201303260504 1530827

[65] Sur S Gleich GJ Swanson MC Bartemes KR Broide DH. Eosinophilic inflammation is associated with elevation of interleukin-5 in the airways of patients with spontaneous symptomatic asthma. J Allergy Clin Immunol (1995) 96(5 Pt 1):661–8. 10.1016/S0091-6749(95)70265-2 7499683

[66] Price DB Rigazio A Campbell JD Bleecker ER Corrigan CJ Thomas M Blood eosinophil count and prospective annual asthma disease burden: a UK cohort study. Lancet Respir Med (2015) 3(11):849–58. 10.1016/S2213-2600(15)00367-7 26493938

[67] Douwes J Gibson P Pekkanen J Pearce N. Non-eosinophilic asthma: importance and possible mechanisms. Thorax (2002) 57(7):643–8. 10.1136/thorax.57.7.643 12096210 PMC1746367

[68] Mejia R Nutman TB. Evaluation and differential diagnosis of marked, persistent eosinophilia. Semin Hematol (2012) 49(2):149–59. 10.1053/j.seminhematol.2012.01.006 22449625 PMC3314223

[69] Simon HU Rothenberg ME Bochner BS Weller PF Wardlaw AJ Wechsler ME Refining the definition of hypereosinophilic syndrome. J Allergy Clin Immunol (2010) 126(1):45–9. 10.1016/j.jaci.2010.03.042 20639008 PMC3400024

[70] Gundel RH Letts LG Gleich GJ. Human eosinophil major basic protein induces airway constriction and airway hyperresponsiveness in primates. J Clin Invest (1991) 87(4):1470–3. 10.1172/JCI115155 2010556 PMC295201

[71] Coyle AJ Ackerman SJ Burch R Proud D Irvin CG. Human eosinophil-granule major basic protein and synthetic polycations induce airway hyperresponsiveness in vivo dependent on bradykinin generation. J Clin Invest (1995) 95(4):1735–40. 10.1172/JCI117850 7706481 PMC295692

[72] Piliponsky AM Gleich GJ Nagler A Bar I Levi-Schaffer F. Non-IgE-dependent activation of human lung- and cord blood-derived mast cells is induced by eosinophil major basic protein and modulated by the membrane form of stem cell factor. Blood (2003) 101(5):1898–904. 10.1182/blood-2002-05-1488 12393403

[73] Ben-Zimra M Bachelet I Seaf M Gleich GJ Levi-Schaffer F Eosinophil major basic protein activates human cord blood mast cells primed with fibroblast membranes by integrin-beta1. Allergy (2013) 68(10):1259–68. 10.1111/all.12232 24112102

[74] Grünig G Warnock M Wakil AE Venkayya R Brombacher F Rennick DM Requirement for IL-13 independently of IL-4 in experimental asthma. Science (1998) 282(5397):2261–3. 10.1126/science.282.5397.2261 9856950 PMC3897229

[75] Hallstrand TS Henderson WR Jr An update on the role of leukotrienes in asthma. Curr Opin Allergy Clin Immunol (2010) 10(1):60–6. 10.1097/ACI.0b013e32833489c3 19915456 PMC2838730

[76] Lee JJ Dimina D Macias MP Ochkur SI McGarry MP O’Neill KR Defining a link with asthma in mice congenitally deficient in eosinophils. Science (2004) 305(5691):1773–6. 10.1126/science.1099472 15375267

[77] Humbles AA Lloyd CM McMillan SJ Friend DS Xanthou G McKenna EE A critical role for eosinophils in allergic airways remodeling. Science (2004) 305(5691):1776–9. 10.1126/science.1100283 15375268

[78] Kay AB The role of eosinophils in the pathogenesis of asthma. Trends Mol Med (2005) 11(4):148–52. 10.1016/j.molmed.2005.02.002 15823751

[79] Leckie MJ ten Brinke A Khan J Diamant Z O’Connor BJ Walls CM Effects of an interleukin-5 blocking monoclonal antibody on eosinophils, airway hyper-responsiveness, and the late asthmatic response. Lancet (2000) 356(9248):2144–8. 10.1016/S0140-6736(00)03496-6 11191542

[80] Frigas E Gleich GJ. The eosinophil and the pathophysiology of asthma. J Allergy Clin Immunol (1986) 77(4):527–37. 10.1016/0091-6749(86)90341-6 3514730

[81] Slifman NR Loegering DA McKean DJ Gleich GJ. Ribonuclease activity associated with human eosinophil-derived neurotoxin and eosinophil cationic protein. J Immunol (1986) 137(9):2913–7. 3760576

[82] Navarro S Aleu J Jimenez M Boix E Cuchillo CM Nogues MV The cytotoxicity of eosinophil cationic protein/ribonuclease 3 on eukaryotic cell lines takes place through its aggregation on the cell membrane. Cell Mol Life Sci (2008) 65(2):324–37. 10.1007/s00018-007-7499-7 18087674 PMC11131711

[83] Durack DT Ackerman SJ Loegering DA Gleich GJ. Purification of human eosinophil-derived neurotoxin. Proc Natl Acad Sci U S A (1981) 78(8):5165–9. 10.1073/pnas.78.8.5165 6946462 PMC320359

[84] Fredens K Dahl R Venge P. The Gordon phenomenon induced by the eosinophil cationic protein and eosinophil protein X. J Allergy Clin Immunol (1982) 70(5):361–6. 10.1016/0091-6749(82)90025-2 7130551

[85] van Dalen CJ Kettle AJ. Substrates and products of eosinophil peroxidase. Biochem J (2001) 358(Pt 1):233–9. 10.1042/0264-6021:3580233 11485572 PMC1222052

[86] Bergeron C Tulic MK Hamid Q. Airway remodelling in asthma: from benchside to clinical practice. Can Respir J (2010) 17(4):e85–93. 10.1155/2010/318029 20808979 PMC2933777

[87] Benayoun L Druilhe A Dombret MC Aubier M Pretolani M. Airway structural alterations selectively associated with severe asthma. Am J Respir Crit Care Med (2003) 167(10):1360–8. 10.1164/rccm.200209-1030OC 12531777

[88] Payne DN Rogers AV Adelroth E Bandi V Guntupalli KK Bush A Early thickening of the reticular basement membrane in children with difficult asthma. Am J Respir Crit Care Med (2003) 167(1):78–82. 10.1164/rccm.200205-414OC 12502479

[89] Torrego A Hew M Oates T Sukkar M Fan Chung K. Expression and activation of TGF-beta isoforms in acute allergen-induced remodelling in asthma. Thorax (2007) 62(4):307–13. 10.1136/thx.2006.063487 17251317 PMC1892798

[90] Phipps S Ying S Wangoo A Ong YE Levi-Schaffer F Kay AB. The relationship between allergen-induced tissue eosinophilia and markers of repair and remodeling in human atopic skin. J Immunol (2002) 169(8):4604–12. 10.4049/jimmunol.169.8.4604 12370399

[91] Ohno I Lea RG Flanders KC Clark DA Banwatt D Dolovich J Eosinophils in chronically inflamed human upper airway tissues express transforming growth factor beta 1 gene (TGF beta 1). J Clin Invest (1992) 89(5):1662–8. 10.1172/JCI115764 1569205 PMC443044

[92] Wong DT Elovic A Matossian K Nagura N McBride J Chou MY Eosinophils from patients with blood eosinophilia express transforming growth factor beta 1. Blood (1991) 78(10):2702–7. 1726708

[93] Ohno I Nitta Y Yamauchi K Hoshi H Honma M Woolley K Transforming growth factor beta 1 (TGF beta 1) gene expression by eosinophils in asthmatic airway inflammation. Am J Respir Cell Mol Biol (1996) 15(3):404–9. 10.1165/ajrcmb.15.3.8810646 8810646

[94] Pégorier S Wagner LA Gleich GJ Pretolani M. Eosinophil-derived cationic proteins activate the synthesis of remodeling factors by airway epithelial cells. J Immunol (2006) 177(7):4861–9. 10.4049/jimmunol.177.7.4861 16982928

[95] Birkland TP Cheavens MD Pincus SH. Human eosinophils stimulate DNA synthesis and matrix production in dermal fibroblasts. Arch Dermatol Res (1994) 286(6):312–8. 10.1007/BF00402221 7979546

[96] Makinde T Murphy RF Agrawal DK. The regulatory role of TGF-beta in airway remodeling in asthma. Immunol Cell Biol (2007) 85(5):348–56. 10.1038/sj.icb.7100044 17325694

[97] Levi-Schaffer F Garbuzenko E Rubin A Reich R Pickholz D Gillery P Human eosinophils regulate human lung- and skin-derived fibroblast properties in vitro: a role for transforming growth factor beta (TGF-beta). Proc Natl Acad Sci U S A (1999) 96(17):9660–5. 10.1073/pnas.96.17.9660 10449750 PMC22266

[98] Vignola AM Chanez P Chiappara G Merendino A Pace E Rizzo A Transforming growth factor-beta expression in mucosal biopsies in asthma and chronic bronchitis. Am J Respir Crit Care Med (1997) 156(2 Pt 1):591–9. 10.1164/ajrccm.156.2.9609066 9279245

[99] Goldsmith AM Bentley JK Zhou L Jia Y Bitar KN Fingar DC Transforming growth factor-beta induces airway smooth muscle hypertrophy. Am J Respir Cell Mol Biol (2006) 34(2):247–54. 10.1165/rcmb.2005-0166OC 16239645 PMC2644185

[100] McMillan SJ Xanthou G Lloyd CM. Manipulation of allergen-induced airway remodeling by treatment with anti-TGF-beta antibody: effect on the Smad signaling pathway. J Immunol (2005) 174(9):5774–80. 10.4049/jimmunol.174.9.5774 15843580

[101] Vignola AM Riccobono L Mirabella A Profita M Chanez P Bellia V Sputum metalloproteinase-9/tissue inhibitor of metalloproteinase-1 ratio correlates with airflow obstruction in asthma and chronic bronchitis. Am J Respir Crit Care Med (1998) 158(6):1945–50. 10.1164/ajrccm.158.6.9803014 9847290

[102] Johnson C Sung HJ Lessner SM Fini ME Galis ZS. Matrix metalloproteinase-9 is required for adequate angiogenic revascularization of ischemic tissues: potential role in capillary branching. Circ Res (2004) 94(2):262–8. 10.1161/01.RES.0000111527.42357.62 14670843 PMC6716372

[103] Hoshino M Takahashi M Aoike N. Expression of vascular endothelial growth factor, basic fibroblast growth factor, and angiogenin immunoreactivity in asthmatic airways and its relationship to angiogenesis. J Allergy Clin Immunol (2001) 107(2):295–301. 10.1067/mai.2001.111928 11174196

[104] Thompson HG Mih JD Krasieva TB Tromberg BJ George SC. Epithelial-derived TGF-beta2 modulates basal and wound-healing subepithelial matrix homeostasis. Am J Physiol Lung Cell Mol Physiol (2006) 291(6):L1277–85. 10.1152/ajplung.00057.2006 16891397

[105] Zagai U Lundahl J Klominek J Venge P Sköld CM. Eosinophil cationic protein stimulates migration of human lung fibroblasts in vitro. Scand J Immunol (2009) 69(4):381–6. 10.1111/j.1365-3083.2009.02233.x 19284504

[106] Hernnäs J Särnstrand B Lindroth P Peterson CG Venge P Malmström A. Eosinophil cationic protein alters proteoglycan metabolism in human lung fibroblast cultures. Eur J Cell Biol (1992) 59(2):352–63. 1493801

[107] Molet S Hamid Q Davoine F Nutku E Taha R Pagé N IL-17 is increased in asthmatic airways and induces human bronchial fibroblasts to produce cytokines. J Allergy Clin Immunol (2001) 108(3):430–8. 10.1067/mai.2001.117929 11544464

[108] Lu S Li H Gao R Gao X Xu F Wang Q IL-17A, but not IL-17F, is indispensable for airway vascular remodeling induced by exaggerated Th17 cell responses in prolonged ovalbumin-challenged mice. J Immunol (2015) 194(8):3557–66. 10.4049/jimmunol.1400829 25780043

[109] Zhu Z Homer RJ Wang Z Chen Q Geba GP Wang J Pulmonary expression of interleukin-13 causes inflammation, mucus hypersecretion, subepithelial fibrosis, physiologic abnormalities, and eotaxin production. J Clin Invest (1999) 103(6):779–88. 10.1172/JCI5909 10079098 PMC408149

[110] Malavia NK Mih JD Raub CB Dinh BT George SC. IL-13 induces a bronchial epithelial phenotype that is profibrotic. Respir Res (2008) 9:27. 10.1186/1465-9921-9-27 18348727 PMC2292179

[111] Wang Q Li H Yao Y Lu G Wang Y Xia D HB-EGF-promoted airway smooth muscle cells and their progenitor migration contribute to airway smooth muscle remodeling in asthmatic mouse. J Immunol (2016) 196(5):2361–7. 10.4049/jimmunol.1402126 26826248

[112] Frossard N Freund V Advenier C Nerve growth factor and its receptors in asthma and inflammation. Eur J Pharmacol (2004) 500(1–3):453–65. 10.1016/j.ejphar.2004.07.044 15464052

[113] Huang LW Sun G Wang DL Kong LF. Inhibition of nerve growth factor/tyrosine kinase receptor A signaling ameliorates airway remodeling in chronic allergic airway inflammation. Eur Rev Med Pharmacol Sci (2015) 19(12):2261–8. 26166652

[114] Henderson WR Jr Chiang GK Tien YT Chi EY. Reversal of allergen-induced airway remodeling by CysLT1 receptor blockade. Am J Respir Crit Care Med (2006) 173(7):718–28. 10.1164/rccm.200501-088OC 16387808 PMC2662952

[115] Kampf C Relova AJ Sandler S Roomans GM. Effects of TNF-alpha, IFN-gamma and IL-beta on normal human bronchial epithelial cells. Eur Respir J (1999) 14(1):84–91. 10.1034/j.1399-3003.1999.14a15.x 10489833

[116] Sullivan DE Ferris M Pociask D Brody AR. Tumor necrosis factor-alpha induces transforming growth factor-beta1 expression in lung fibroblasts through the extracellular signal-regulated kinase pathway. Am J Respir Cell Mol Biol (2005) 32(4):342–9. 10.1165/rcmb.2004-0288OC 15653932

[117] Halwani R Vazquez-Tello A Sumi Y Pureza MA Bahammam A Al-Jahdali H Eosinophils induce airway smooth muscle cell proliferation. J Clin Immunol (2013) 33(3):595–604. 10.1007/s10875-012-9836-3 23180361

[118] Fanat AI Thomson JV Radford K Nair P Sehmi R. Human airway smooth muscle promotes eosinophil differentiation. Clin Exp Allergy (2009) 39(7):1009–17. 10.1111/j.1365-2222.2009.03246.x 19438586

[119] Flood-Page P Menzies-Gow A Phipps S Ying S Wangoo A Ludwig MS Anti-IL-5 treatment reduces deposition of ECM proteins in the bronchial subepithelial basement membrane of mild atopic asthmatics. J Clin Invest (2003) 112(7):1029–36. 10.1172/JCI17974 14523040 PMC198522

[120] Pizzichini MM Pizzichini E Clelland L Efthimiadis A Pavord I Dolovich J Prednisone-dependent asthma: inflammatory indices in induced sputum. Eur Respir J (1999) 13(1):15–21. 10.1183/09031936.99.13101599 10836317

[121] Green RH Brightling CE McKenna S Hargadon B Parker D Bradding P Asthma exacerbations and sputum eosinophil counts: a randomised controlled trial. Lancet (2002) 360(9347):1715–21. 10.1016/S0140-6736(02)11679-5 12480423

[122] Haldar P Brightling CE Hargadon B Gupta S Monteiro W Sousa A Mepolizumab and exacerbations of refractory eosinophilic asthma. N Engl J Med (2009) 360(10):973–84. 10.1056/NEJMoa0808991 19264686 PMC3992367

[123] Pavord ID Korn S Howarth P Bleecker ER Buhl R Keene ON Mepolizumab for severe eosinophilic asthma (DREAM): a multicentre, double-blind, placebo-controlled trial. Lancet (2012) 380(9842):651–9. 10.1016/S0140-6736(12)60988-X 22901886

[124] Ortega HG Liu MC Pavord ID Brusselle GG FitzGerald JM Chetta A Mepolizumab treatment in patients with severe eosinophilic asthma. N Engl J Med (2014) 371(13):1198–207. 10.1056/NEJMoa1403290 25199059

[125] Castro M Zangrilli J Wechsler ME Bateman ED Brusselle GG Bardin P Reslizumab for inadequately controlled asthma with elevated blood eosinophil counts: results from two multicentre, parallel, double-blind, randomised, placebo-controlled, phase 3 trials. Lancet Respir Med (2015) 3(5):355–66. 10.1016/S2213-2600(15)00042-9 25736990

[126] Bel EH Wenzel SE Thompson PJ Prazma CM Keene ON Yancey SW Oral glucocorticoid-sparing effect of mepolizumab in eosinophilic asthma. N Engl J Med (2014) 371(13):1189–97. 10.1056/NEJMoa1403291 25199060

[127] Kolbeck R Kozhich A Koike M Peng L Andersson CK Damschroder MM MEDI-563, a humanized anti-IL-5 receptor alpha mAb with enhanced antibody-dependent cell-mediated cytotoxicity function. J Allergy Clin Immunol (2010) 125(6):1344–53.e2. 10.1016/j.jaci.2010.04.004 20513525

[128] Laviolette M Gossage DL Gauvreau G Leigh R Olivenstein R Katial R Effects of benralizumab on airway eosinophils in asthmatic patients with sputum eosinophilia. J Allergy Clin Immunol (2013) 132(5):1086–96.e5. 10.1016/j.jaci.2013.05.020 23866823 PMC4172321

[129] FitzGerald JM Bleecker ER Nair P Korn S Ohta K Lommatzsch M Benralizumab, an anti-interleukin-5 receptor alpha monoclonal antibody, as add-on treatment for patients with severe, uncontrolled, eosinophilic asthma (CALIMA): a randomised, double-blind, placebo-controlled phase 3 trial. Lancet (2016) 388(10056):2128–41. 10.1016/S0140-6736(16)31322-8 27609406

[130] Bleecker ER FitzGerald JM Chanez P Papi A Weinstein SF Barker P Efficacy and safety of benralizumab for patients with severe asthma uncontrolled with high-dosage inhaled corticosteroids and long-acting beta2-agonists (SIROCCO): a randomised, multicentre, placebo-controlled phase 3 trial. Lancet (2016) 388(10056):2115–27. 10.1016/S0140-6736(16)31324-1 27609408

[131] Shenoy NG Gleich GJ Thomas LL. Eosinophil major basic protein stimulates neutrophil superoxide production by a class IA phosphoinositide 3-kinase and protein kinase C-zeta-dependent pathway. J Immunol (2003) 171(7):3734–41. 10.4049/jimmunol.171.7.3734 14500673

[132] Kannan Y Ushio H Koyama H Okada M Oikawa M Yoshihara T 2.5S nerve growth factor enhances survival, phagocytosis, and superoxide production of murine neutrophils. Blood (1991) 77(6):1320–5. 1848116

[133] Kawamoto K Okada T Kannan Y Ushio H Matsumoto M Matsuda H. Nerve growth factor prevents apoptosis of rat peritoneal mast cells through the trk proto-oncogene receptor. Blood (1995) 86(12):4638–44. 8541555

[134] Yang D Chen Q Rosenberg HF Rybak SM Newton DL Wang ZY Human ribonuclease A superfamily members, eosinophil-derived neurotoxin and pancreatic ribonuclease, induce dendritic cell maturation and activation. J Immunol (2004) 173(10):6134–42. 10.4049/jimmunol.173.10.6134 15528350 PMC2847482

[135] Akuthota P Wang H Weller PF. Eosinophils as antigen-presenting cells in allergic upper airway disease. Curr Opin Allergy Clin Immunol (2010) 10(1):14–9. 10.1097/ACI.0b013e328334f693 19949323 PMC2865844

[136] Shi HZ Humbles A Gerard C Jin Z Weller PF. Lymph node trafficking and antigen presentation by endobronchial eosinophils. J Clin Invest (2000) 105(7):945–53. 10.1172/JCI8945 10749574 PMC377484

[137] Cocks BG de Waal Malefyt R Galizzi JP de Vries JE Aversa G. IL-13 induces proliferation and differentiation of human B cells activated by the CD40 ligand. Int Immunol (1993) 5(6):657–63. 10.1093/intimm/5.6.657 7688562

[138] Geha RS Jabara HH Brodeur SR The regulation of immunoglobulin E class-switch recombination. Nat Rev Immunol (2003) 3(9):721–32. 10.1038/nri1181 12949496

[139] Chu VT Fröhlich A Steinhauser G Scheel T Roch T Fillatreau S Eosinophils are required for the maintenance of plasma cells in the bone marrow. Nat Immunol (2011) 12(2):151–9. 10.1038/ni.1981 21217761

[140] Jourdan M Cren M Robert N Bolloré K Fest T Duperray C IL-6 supports the generation of human long-lived plasma cells in combination with either APRIL or stromal cell-soluble factors. Leukemia (2014) 28(8):1647–56. 10.1038/leu.2014.61 24504026

[141] Liu LY Bates ME Jarjour NN Busse WW Bertics PJ Kelly EA. Generation of Th1 and Th2 chemokines by human eosinophils: evidence for a critical role of TNF-alpha. J Immunol (2007) 179(7):4840–8. 10.4049/jimmunol.179.7.4840 17878383

[142] Odemuyiwa SO Ghahary A Li Y Puttagunta L Lee JE Musat-Marcu S Cutting edge: human eosinophils regulate T cell subset selection through indoleamine 2,3-dioxygenase. J Immunol (2004) 173(10):5909–13. 10.4049/jimmunol.173.10.5909 15528322

[143] Xu H Zhang GX Ciric B Rostami A. IDO: a double-edged sword for T(H)1/T(H)2 regulation. Immunol Lett (2008) 121(1):1–6. 10.1016/j.imlet.2008.08.008 18824197 PMC2628165

[144] Ravin KA Loy M The eosinophil in infection. Clin Rev Allergy Immunol (2016) 50(2):214–27. 10.1007/s12016-015-8525-4 26690368

[145] Hogan SP Rosenberg HF Moqbel R Phipps S Foster PS Lacy P Eosinophils: biological properties and role in health and disease. Clin Exp Allergy (2008) 38(5):709–50. 10.1111/j.1365-2222.2008.02958.x 18384431

[146] Rosenberg HF Domachowske JB Eosinophils, eosinophil ribonucleases, and their role in host defense against respiratory virus pathogens. J Leukoc Biol (2001) 70(5):691–8. 11698487

[147] Torrent M Navarro S Moussaoui M Nogués MV Boix E. Eosinophil cationic protein high-affinity binding to bacteria-wall lipopolysaccharides and peptidoglycans. Biochemistry (2008) 47(11):3544–55. 10.1021/bi702065b 18293932

[148] Ackerman SJ Gleich GJ Loegering DA Richardson BA Butterworth AE. Comparative toxicity of purified human eosinophil granule cationic proteins for schistosomula of *Schistosoma mansoni*. Am J Trop Med Hyg (1985) 34(4):735–45. 10.4269/ajtmh.1985.34.735 4025686

[149] Domachowske JB Dyer KD Bonville CA Rosenberg HF. Recombinant human eosinophil-derived neurotoxin/RNase 2 functions as an effective antiviral agent against respiratory syncytial virus. J Infect Dis (1998) 177(6):1458–64. 10.1086/515322 9607820

[150] Acharya KR Ackerman SJ. Eosinophil granule proteins: form and function. J Biol Chem (2014) 289(25):17406–15. 10.1074/jbc.R113.546218 24802755 PMC4067173

[151] Yazdanbakhsh M Eckmann CM Bot AA Roos D. Bactericidal action of eosinophils from normal human blood. Infect Immun (1986) 53(1):192–8. 3522428 10.1128/iai.53.1.192-198.1986 PMC260096

[152] Yousefi S Gold JA Andina N Lee JJ Kelly AM Kozlowski E Catapult-like release of mitochondrial DNA by eosinophils contributes to antibacterial defense. Nat Med (2008) 14(9):949–53. 10.1038/nm.1855 18690244

[153] Zeck-Kapp G Kroegel C Riede UN Kapp A. Mechanisms of human eosinophil activation by complement protein C5a and platelet-activating factor: similar functional responses are accompanied by different morphologic alterations. Allergy (1995) 50(1):34–47. 10.1111/j.1398-9995.1995.tb02481.x 7741187

[154] DiScipio RG Schraufstatter IU. The role of the complement anaphylatoxins in the recruitment of eosinophils. Int Immunopharmacol (2007) 7(14):1909–23. 10.1016/j.intimp.2007.07.006 18039528

[155] Shin EH Osada Y Sagara H Takatsu K Kojima S. Involvement of complement and fibronectin in eosinophil-mediated damage to *Nippostrongylus brasiliensis* larvae. Parasite Immunol (2001) 23(1):27–37. 10.1046/j.1365-3024.2001.00352.x 11136475

[156] Giacomin PR Gordon DL Botto M Daha MR Sanderson SD Taylor SM The role of complement in innate, adaptive and eosinophil-dependent immunity to the nematode *Nippostrongylus brasiliensis*. Mol Immunol (2008) 45(2):446–55. 10.1016/j.molimm.2007.05.029 17675237

[157] Porter PC Lim DJ Maskatia ZK Mak G Tsai CL Citardi MJ Airway surface mycosis in chronic TH2-associated airway disease. J Allergy Clin Immunol (2014) 134(2):325–31. 10.1016/j.jaci.2014.04.028 24928648 PMC4119856

[158] Papadopoulos NG Stanciu LA Papi A Holgate ST Johnston SL. A defective type 1 response to rhinovirus in atopic asthma. Thorax (2002) 57(4):328–32. 10.1136/thorax.57.4.328 11923551 PMC1746310

[159] Wark PA Johnston SL Bucchieri F Powell R Puddicombe S Laza-Stanca V Asthmatic bronchial epithelial cells have a deficient innate immune response to infection with rhinovirus. J Exp Med (2005) 201(6):937–47. 10.1084/jem.20041901 15781584 PMC2213100

[160] Mathur SK Fichtinger PS Kelly JT Lee WM Gern JE Jarjour NN. Interaction between allergy and innate immunity: model for eosinophil regulation of epithelial cell interferon expression. Ann Allergy Asthma Immunol (2013) 111(1):25–31. 10.1016/j.anai.2013.05.010 23806456 PMC3708694

[161] Charlson ES Bittinger K Haas AR Fitzgerald AS Frank I Yadav A Topographical continuity of bacterial populations in the healthy human respiratory tract. Am J Respir Crit Care Med (2011) 184(8):957–63. 10.1164/rccm.201104-0655OC 21680950 PMC3208663

[162] Hilty M Burke C Pedro H Cardenas P Bush A Bossley C Disordered microbial communities in asthmatic airways. PLoS One (2010) 5(1):e8578. 10.1371/journal.pone.0008578 20052417 PMC2798952

[163] Sverrild A Kiilerich P Brejnrod A Pedersen R Porsbjerg C Bergqvist A Eosinophilic airway inflammation in asthmatic patients is associated with an altered airway microbiome. J Allergy Clin Immunol (2016). 10.1016/j.jaci.2016.10.046 28042058

